# Association of V_H_4-59 Antibody Variable Gene Usage with Recognition of an Immunodominant Epitope on the HIV-1 Gag Protein

**DOI:** 10.1371/journal.pone.0133509

**Published:** 2015-07-30

**Authors:** Valentine U. Chukwuma, Mark D. Hicar, Xuemin Chen, Katherine J. Nicholas, Amanda Joyner, Spyros A. Kalams, Gary Landucci, Donald N. Forthal, Paul W. Spearman, James E. Crowe

**Affiliations:** 1 Department of Pathology, Microbiology and Immunology, Vanderbilt University, Nashville, Tennessee, United States of America; 2 Department of Pediatrics, Vanderbilt University, Nashville, Tennessee, United States of America; 3 Department of Medicine, Vanderbilt University, Nashville, Tennessee, United States of America; 4 Vanderbilt Vaccine Center, Microbiology and Immunology, Vanderbilt University, Nashville, Tennessee, United States of America; 5 Departments of Pediatrics, Microbiology and Immunology, Emory University School of Medicine, Atlanta, Georgia, United States of America; 6 Children’s Healthcare of Atlanta, Atlanta, Georgia, United States of America; 7 Department of Medicine, University of California Irvine, Irvine, California, United States of America; New York State Dept. Health, UNITED STATES

## Abstract

The human antibody response against HIV-1 infection recognizes diverse antigenic subunits of the virion, and includes a high level of antibodies to the Gag protein. We report here the isolation and characterization of a subset of Gag-specific human monoclonal antibodies (mAbs) that were prevalent in the antibody repertoire of an HIV-infected individual. Several lineages of Gag-specifc mAbs were encoded by a single antibody heavy chain variable region, V_H_4-59, and a representative antibody from this group designated mAb 3E4 recognized a linear epitope on the globular head of the p17 subunit of Gag. We found no evidence that mAb 3E4 exhibited any function in laboratory studies aimed at elucidating the immunologic activity, including assays for neutralization, Ab-dependent cell-mediated virus inhibition, or enhanced T cell reactivity caused by Gag-3E4 complexes. The findings suggest this immunodominant epitope in Gag protein, which is associated with V_H_4-59 germline gene usage, may induce a high level of B cells that encode binding but non-functional antibodies that occupy significant repertoire space following HIV infection. The studies define an additional specific molecular mechanism in the immune distraction activity of the HIV virion.

## Introduction

The emergence of neutralizing antibody responses to HIV-1 in naturally infected humans is delayed compared to the induction of comparable antibodies following most other viral infections. The basis for the slow kinetics of this HIV-1 neutralizing response is understood incompletely [1]. One contributor to this delayed neutralizing response is the large number of highly antigenic epitopes that induce high titers of binding antibodies that are unable to neutralize infectious virus [2]. Many of these binding antibody epitopes are found in variable loops on the HIV-1 envelope (Env) glycoprotein, or on conformationally altered or incomplete forms of the glycoprotein [3]. Infected subjects also make non-neutralizing antibodies to HIV-1 internal proteins [4], most commonly those specified by the *gag* gene, which encodes a polyprotein precursor pr55 protein that is cleaved into several core proteins, including the p24 capsid and p17 Matrix proteins [5].

HIV-1 Gag protein plays several roles during the life cycle of the virus, including assembly, maturation and particle release [6–7]. The Gag protein is sufficient for the production of non-infectious virus-like particles (VLPs), even in the absence of other viral proteins [8]. These HIV VLPs are composed of a viral core surrounded by a lipid membrane derived from the host cell, which also contains host proteins [9]. The C-terminus of the Gag protein is the p17 Matrix protein, which has a number of important functional features. Deletion of this region abrogates VLP formation, indicating that p17 is critical for VLP assembly [10]. In addition, p17 is involved in immunological processes, such as enhancing HIV-1 infection by promoting rapid proliferation of IL-2 stimulated peripheral blood mononuclear cells [11] and has been shown to up-regulate the secretion of IFN-γ and TNF-α through interactions with a specific receptor on activated T cells [12]. Notably, by interacting with B cells and triggering intracellular signaling pathways, p17 protein has also been shown to play a role in AIDS-associated lymphoma [13].

Monoclonal antibodies (mAbs) against Gag and p17 proteins have been isolated from vaccinated animals [14], and HIV-1 infected subjects have been reported to possess high plasma titers of Gag and p17 antibodies [15]. Plasma antibodies directed against p17 have been associated with neutralizing antibody titers, and have been shown to bind infected cell surfaces [16], suggesting that p17 might be exposed on the surface of the HIV-1 virion in some cases [17]. Also, high titers of antibodies against p17 correlate with slower progression to AIDS [18]. Several epitopes that are conserved across HIV-1, HIV-2 and SIV, are located in the Gag protein [19], and overlapping oligopeptides have been used to map the epitopes of some anti-Gag monoclonal antibodies [20].

In this report, we describe the isolation and characterization of a panel of mAbs from an HIV-1 infected subject that reacted to Gag-only VLPs that were not expressing envelope. Notably, these mAbs shared the use of the heavy chain V_H_4-59 antibody variable gene. We identified the epitope of these mAbs on the surface of the p17 protein, and performed functional studies to test the immunological relevance of this immunodominant response.

## Materials and Methods

### Ethics statement

The study was approved by the Vanderbilt University Institutional Review Board, and the study participants provided written informed consent. The data were analyzed anonymously.

### Antibody variable gene sequences

Single human B cells from an HIV-infected subject (designated Vanderbilt HIV cohort subject 10076) were isolated by flow cytometric sorting following surface labeling with HIV virus-like particles (VLPs) containing green fluorescent protein. The single B cells were expanded in culture and shown to bind to HIV VLPs generated in a recombinant system using Gag protein in the absence of HIV Env. The antibody gene sequences for each of the Gag-only particle-binding clones was identified by RNA extraction, RT-PCR with antibody variable gene primers, and cloned in a TA plasmid vector, followed by automated sequence analysis of the insert. The antibody sequences obtained were analyzed using an online analysis tool (IMGT; http://www.imgt.org), which revealed that each of these six single-cell derived clones expressed antibodies encoded by the V_H_4-59 heavy chain gene segment. We obtained the light chain gene for the mAb 3E4 in a similar fashion.

### Expression and purification of V_H_4-59 monoclonal antibodies (mAbs)

We reasoned that since the six antibodies from a single donor each used the V_H_4-59 heavy chain gene segment and bound to Gag-only VLPs, it was likely they all bound to the same epitope in a manner that was driven principally by heavy chain interactions. Therefore, we used an expression strategy in which the 3E4 light chain was paired independently with each of the six heavy chains for recombinant expression. To prepare the V_H_4-59 IgG1 proteins, we used recombinant expression in mammalian cells, as previously described [21]. Briefly, the six V_H_4-59 gene-segment-encoded antibody heavy-chain genes were synthesized and each cloned into the pConIgG1Sma vector, and the mAb 3E4 light-chain gene was cloned into the pConK2Sma vector (Lonza). Plasmids encoding full-length IgG incorporating one of the V_H_4-59 gene segments and the plasmid with the 3E4 light chain gene each were transformed into DH5α *E*. *coli* cells for EndoFree Plasmid Maxi DNA preparation (Sigma), after sequence verification of the insert. A plasmid encoding the mAb 3E4 light chain was used for expression of each of the six V_H_4-59 gene-segment-encoded mAbs. Plasmids encoding heavy- and light-chain DNAs were co-transfected transiently into HEK 293 F cells (Invitrogen) using Polyfect reagent (Qiagen), and the cells were incubated at 37^°^C in humidified air with 8% CO_2_ in shaker flasks. Supernatant was harvested after 1 week, fast-performance liquid chromatography (FPLC) purified with protein G columns (GE), and concentrated with Amicon Ultra centrifugal filters with a 30-kDa molecular mass cutoff (Millipore).

### Production of HIV virus-like-particles

Particles incorporating HIV Env protein (Env VLPs) or lacking Env protein (Gag-only VLPs) were generated by recombinant techniques. Briefly, cryopreserved inducible T-Rex 293 cells [22] expressing Env or Gag-only VLPs were resurrected and grown through two rounds of expansion in growth medium (DMEM containing 10% TET-Free Fetal Bovine Serum, 1% penicillin-streptomycin solution and 4 mM L-glutamine) in T150 tissue culture flasks. On the third round of expansion, cells were grown in selection medium (growth medium containing hygromycin [100 μg/mL], zeocin [100 μg/mL], blastacidin [5 μg/mL] and puromycin [5 μg/mL]). When the cells were 95% confluent, the medium was aspirated and the cells were induced by the addition of fresh selection medium containing doxycycline [2 μg/mL], and incubated at 37°C for 48 hours. After 48 hours, the supernatant was centrifuged at 3,000 rpm in a tabletop centrifuge for 10 minutes and filtered through a 0.45-micron filter. The flow-through was passed through a sucrose gradient (20% sucrose w/v in DPBS) by ultracentrifugation at 100,000 × *g* for 2 hours. The top layer of medium was aspirated, and the sucrose cushion poured out slowly. Residual medium and sucrose around the pellet were dried using a KimWipe tissue. The pellet was re-suspended in 100–200 μL of cold DPBS and placed on ice for two minutes before transfer into a cryovial for long-term storage at -80^°^C. Working stocks of VLPs were kept at 4^°^C.

### Production of monomeric recombinant HIV gp120 protein

Monomeric recombinant HIV-1 gp120 protein suspensions were produced by transiently transfecting plasmids encoding HIV-1 subtype B BAL gp120 (GenBank No. M68893) in HEK Freestyle 293 F cells, followed by incubation at 37°C and 8% CO_2_ on an orbital shaker for 7 days, after which the supernatant was collected and clarified by centrifugation at 400 × *g* for 10 minutes at 4°C. The supernatant then was filtered through a 0.2-micron filter, and the recombinant gp120 was purified using a 5 mL HisTrap HP column (GE Healthcare) on an AKTA FPLC (Amersham Biosciences) following the manufacturer’s instructions. Fractions were collected and concentrated using 100 KD centrifugal columns (Millipore).

### Enzyme linked immunosorbent assays (ELISA) with virus-like particles or proteins

For detection of antibody binding to HIV virus-like particles (Env VLP or Gag-only VLP) or to monomeric recombinant HIV gp120 protein, Immulon II HB 96-well microplates (Dynex Technologies) were coated with 100 μL of Env VLP, Gag-only VLP diluted 1:200 in D-PBS, or monomeric gp120 protein diluted 1:5,000 in PBS, and stored overnight at room temperature. The plates were blocked for 2 h at 4°C with 200 μL per well of D-PBS containing 10% (v/v) FBS. We added 100 μL of antibody at a concentration of 1 μg/mL in diluent (7.5% FBS, 0.05% Tween-20 in D-PBS) to individual wells, incubated the plate for 1 h at 37°C, washed, and detected binding with 100 μL of HRP-conjugated goat anti-human IgG (Southern Biotechnology Associates) diluted 1:1,000 in diluent. The plates were washed, and 100 μL of TMB substrate (Pierce) was added to individual wells, and 50 μL of 1N HCL was added to each well to stop the reaction. The optical density of solutions in the plates was read at 450 nm using a Spectramax M5 plate reader (Molecular Devices).

### Western blot analysis

HEK 293 T cells, TZM-bl cells, HEK 293 Freestyle cells or Jurkat cells were washed with 1x D-PBS and lysed with 1% Triton x 100 lysis buffer [Triton-X 100, 1X D-PBS pH 7.4, 2M MgCl_2_, Protease Inhibitor Cocktail (Sigma), and 200 mM phenylmethanesulfonylfluoride]. Cell lysates were ultracentrifuged at 13,000 rpm for 10 minutes at 4^°^C, and the supernatants were collected as cell lysates. Cell lysates, Env VLP and Gag-only VLP were subjected to SDS-polyacrylamide gel electrophoresis and transferred to a nitrocellulose membrane (Invitrogen). The membrane was blocked with Odyssey block (LI-COR) diluted 1:1 with D-PBS for 1 h at room temperature. After blocking, the membrane was incubated with mAb 3E4 (final concentration 250 ng/mL in Odyssey block diluted 1:1 with D-PBS-T) for 1 h at room temperature followed by four washes in D-PBS-T for 5 min each. The membrane was incubated with the IRDye 800CW Goat anti-Human IgG (H+L) (LI-COR) (1:5,000 dilution) for 1 hour at room temperature followed by four washes in D-PBS-T for 5 min each, and two subsequent washes in 1X D-PBS. The image was acquired using Odyssey infrared imaging system (LI-COR).

### Peptide ELISA

For detection of antibody binding to HIV peptides, Immulon II HB 96-well microplates (Dynex Technologies, Chantilly, VA) were coated with 50 μL per well of peptide diluted to 10 μg/mL in solubility buffer [50% (water + 0.1% trifluoroacetic acid) + 50% (acetonitrile + 0.075% trifluoroacetic acid)] and incubated overnight at 4°C. Plates were washed six times with PBS-T (PBS with 0.05% Tween-20) and blocked for 2 h at room temperature with 150 μL per well of D-PBS containing 10% (v/v) FBS. Plates were washed six times with PBS-T. 50 μL of MAb 3E4 at a concentration of 250 ng/mL was added to each well and the plates were incubated overnight at 4°C. Plates were washed six times with PBS-T. 50 μL of alkaline phosphatase conjugated goat anti-human IgG (Sigma) diluted 1:10,000 in diluent (7.5% FBS, 0.05% Tween-20 in D-PBS) was added to each well and the plates were incubated for 2 h at room temperature. Plates were washed six times with PBS-T. 50 μL of p-nitrophenyl phosphate (Sigma) (1 tablet dissolved per 5 mL in sodium carbonate buffer) was added to each well and the plates were incubated at room temperature for 15–30 minutes. The optical density of solutions in the plates was read at 405 nm using a Spectramax M5 plate reader (Molecular Devices).

### Matrix protein mapping

Each member of a panel of HXB2 Matrix (MA) mutants from the laboratory of Max Essex [23] was cloned by PCR using Nco1-BamH1 sites into the pTM1 vector, using a similar strategy as we previously reported [24], but employing a BamH1 primer at the 3′ end of the Pr55Gag coding sequence instead of at the end of MA. This strategy created a library of MA deletions in the context of Pr55Gag termed p55MAD 1–10, instead of the deletions of MA alone. The nucleic acid sequences of each deletion construct were confirmed by Sanger sequencing. Plasmids were transfected individually into HeLa cells and simultaneously infected with VTF7-3 for expression of T7 polymerase. Cell lysates were harvested for loading on SDS-PAGE, and the resulting gel probed with CA-183 (to verify protein expression) or with mAb 3E4 by western blotting. The LiCor Odyssey instrument was used for detection of specific reactivity.

### Confocal microscopic imaging of antibody binding to mature or immature HIV particles

Mature or immature HIV-1 virions were plated on poly-D-lysine coated dishes (MatTek). Virions were incubated with Env-specific mAb b12 or Gag-specific mAb 3E4 for two h at room temperature, fixed in 2% paraformaldehyde for 15 min at room temperature, washed five times with PBS, incubated with a Cy5-conjugated anti-human IgG (Jackson ImmunoResearch Laboratories, Inc.) at a concentration of 14 μg/mL for one hour at room temperature, washed five times with PBS, mounted, and imaged using an LSM 510 META inverted confocal microscope (Zeiss). Image acquisition was performed using a 63× objective lens with 2× optical zoom with line averaging for a 1024×1024 pixel image. GFP imaging was performed with an excitation wavelength of 488 nm and band pass 505–550 emission filter. Cy5 imaging was performed with an excitation wavelength of 633 nm and a long pass 650-emission filter.

### Neutralization assay

Neutralization assays were performed using the tier 1 clade B SF162 pseudovirus in the TZM-bl cell-based assay. Two-fold serial dilutions of mAb 3E4 were incubated for 90 min at 37°C in the presence of single-round-competent virions. The virus-mAb mixture was added for 48 h at 37°C to TZM-bl cells (ATCC, catalog number PTA-5659) plated at a density of 1 x 10^4^ cells per well in a 96-well plate. 100 μL of the cell supernatants were removed, and 100 μL of BrightGlo (Promega) was added to each well. Plates were incubated for 2 min at room temperature with gentle shaking to lyse the cells. 150 μL of the lysate was transferred to a microtiter plate, and the cell-associated luciferase activity (luminescence) for each well was determined on a Modulus microplate reader (Promega).

### T cell reactivity assays in the presence or absence of matrix antibodies

T cell reactivity assays were performed as previously described [25]. Briefly, peripheral blood mononuclear cells (PBMCs) from treatment-naïve HIV+ donors were separated from blood sample using a Ficoll-Paque Plus density gradient, cryopreserved, and stored in liquid nitrogen. PBMC from 4 donors with known Gag responses were thawed and cultured in R10 medium (RPMI 1640 containing 10% heat inactivated FCS, 2 mM L-glutamine, 50 μg/mL penicillin, 50 μg/mL streptomycin, and 10mM HEPES buffer) overnight at 37°C with 5% CO_2_ under a variety of conditions. All cells were co-stimulated with anti-CD28 and anti-CD49d (1 μg/mL each, from Becton Dickinson [BD]). A subset of cells was cultured only in the co-stimulated medium, while others were stimulated with 10 μg/mL HIV-1_IIIB_ pr55 Gag (NIH AIDS Reagent Program) with or without the 3E4 mAb (10 μg/mL). After overnight incubation, the cells were washed in PBS and incubated first with a viability dye (LIVE/DEAD Aqua, Life Technologies) and then stained with a combination of monoclonal antibodies: CD3-AF700 (UCHT1; BD), CD14-V500 (M5E2; BD), CD19-V500 (HIB19; BD), CD4-PETR (S3.5; Invitrogen), CD8-APCA750 (3B5; Invitrogen), HLA-DR-FITC (L243; BD), CD69-APC (FN50; BD), and CD25-PE (M-A251; BD). Samples then were fixed, analyzed on a BD FACSAria cytometer, and evaluated using BD Biosciences FACSDiva Software. Dead cells and cells that were CD14+ or CD19+ were excluded from analysis. CD4+ and CD8+ T cells then were evaluated for dual CD69 and CD25 expression.

### Antibody-dependent cell-mediated viral inhibition (ADCVI)

The ADCVI assay was conducted using methods similar to those previously described [26]. Briefly, target cells for the ADCVI assay were prepared by inoculating polybrene-treated CEM.NKR-CCR5 cells (National Institutes of Health AIDS Research and Reference Program) with a clinical strain of HIV-1 (HIV-192US657) at a multiplicity of infection of 0.05. After 48 h, target cells were washed to remove cell-free virus. Effector cells (PBMCs from healthy donors) were added to target cells at an E:T ratio of 10:1. Monoclonal antibodies were added to target and effector cells to achieve a final concentration of 100 μg/mL. All wells were washed (5x) on day three to remove supernatants with mAbs and refed with complete medium. Four days later, supernatant fluid was collected, and p24 was measured by ELISA (Zeptometrix, Buffalo, NY). The percent inhibition due to ADCVI was calculated relative to an HIV-negative control as follows: percent inhibition = 100(1 –[(p24p)/(p24n)]), where (p24p) and (p24n) are concentrations of p24 in supernatant fluid from wells containing a source of HIV-positive or HIV-negative Ab, respectively. Individual mAbs were assayed in triplicate using cells from a single effector cell donor. Each mAb was assayed in two separate assays with different donors, and the mean percent inhibition was calculated.

## Results

### Isolation of HIV-specific single human B cells

**Fig 1:**
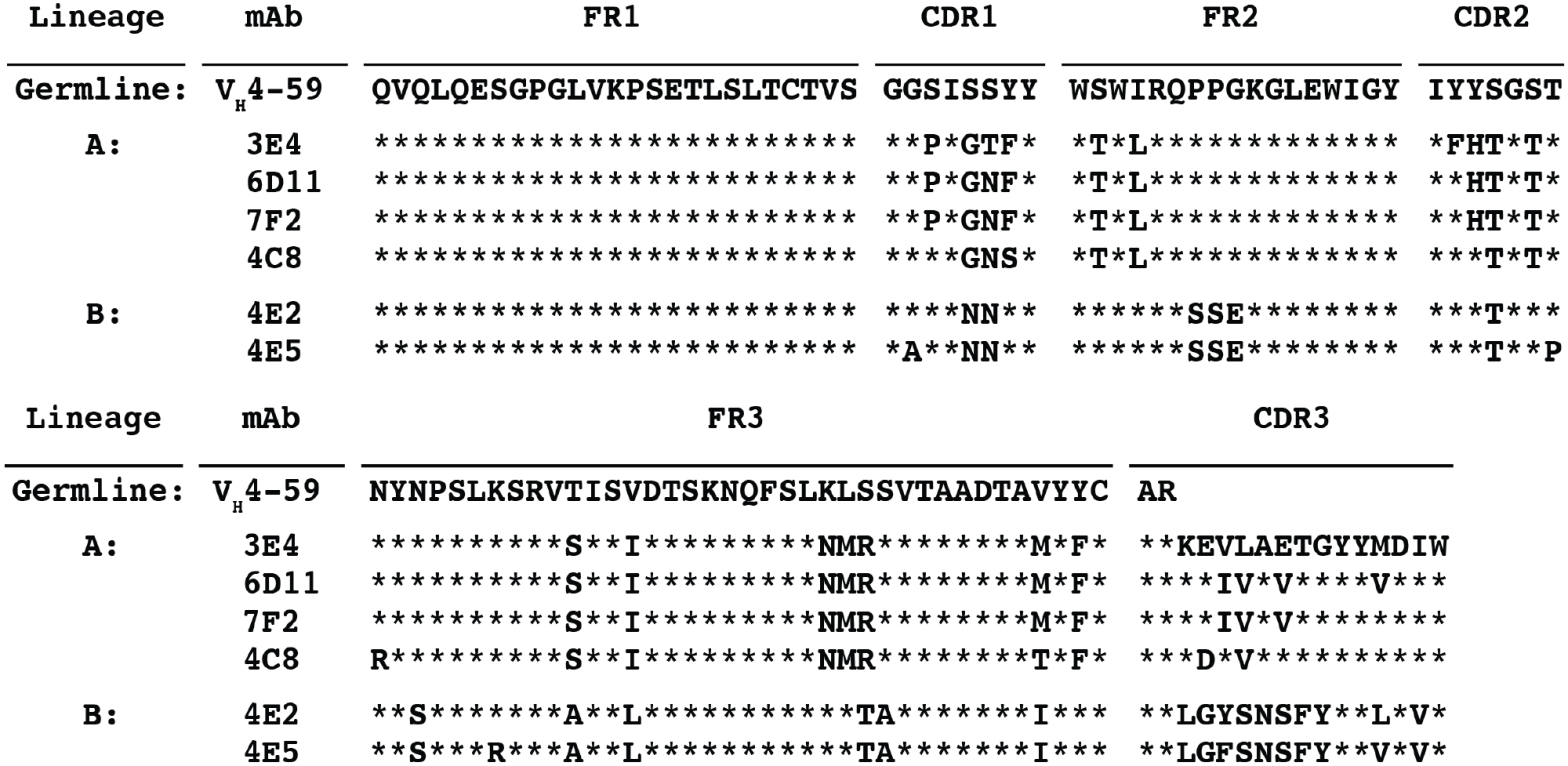
Heavy chain amino acid sequence alignment of V_H_4-59 encoded mAbs. The heavy chain amino acid sequence of HIV-specific V_H_4-59 encoded mAbs are shown aligned to the V_H_4-59 germline gene. Conservation with the germline gene sequence is indicated by a * symbol in the alignments.

We performed single cell cytometric sorting of B cells from an HIV-infected individual designated as subject 10076, using fluorescently labeled VLPs displaying a stabilized form of the BaL strain HIV Env protein. Out of 448 B cells sorted, 45 B cells were shown to be secreting HIV-specific antibodies in culture. Through binding analysis of B cell supernates to HIV antigens, we identified a subset of six B cells that showed binding to HIV VLPs, but not to purified HIV Env proteins. This subset of B cells represented a dominant response to HIV infection this individual, and sequence analysis revealed that the six mAbs with this binding pattern were encoded by the same heavy chain variable germline gene, V_H_4-59 (Table 1). Comparison of the heavy chain amino acid sequences to the germline gene sequence and to each other showed that the mAbs grouped into two distinct lineages, designated lineages A and B. Further analysis showed that although mAbs 3E4, 4C8, 6D11 and 7F2 shared sequence homology within the framework regions, there were distinguishing mutations occurring in the complementarity determining regions particularly in the HCDR3 of 3E4 (Fig 1).

**Table 1:** Heavy chain gene analysis of Gag-only VLP binding antibodies.

mAb	V_H_ gene and allele	D gene and allele	J_H_ gene and allele	Number of somatically mutated nucleotides
3E4	V_H_4-59*01 F	D2-21*01 F	J_H_6*03 F	17
4C8	V_H_4-59*01 F	D2-21*01 F	J_H_6*03 F	15
6D11	V_H_4-59*01 F	D5-12*01 F	J_H_6*03 F	16
7F2	V_H_4-59*01 F	D5-12*01 F	J_H_6*03 F	16
4E2	V_H_4-59*01 F	D6-13*01 F	J_H_6*03 F	13
4E5	V_H_4-59*01 F	D6-13*01 F	J_H_6*03 F	16

### Binding of V_H_4-59-encoded antibodies to HIV antigens

**Fig 2:**
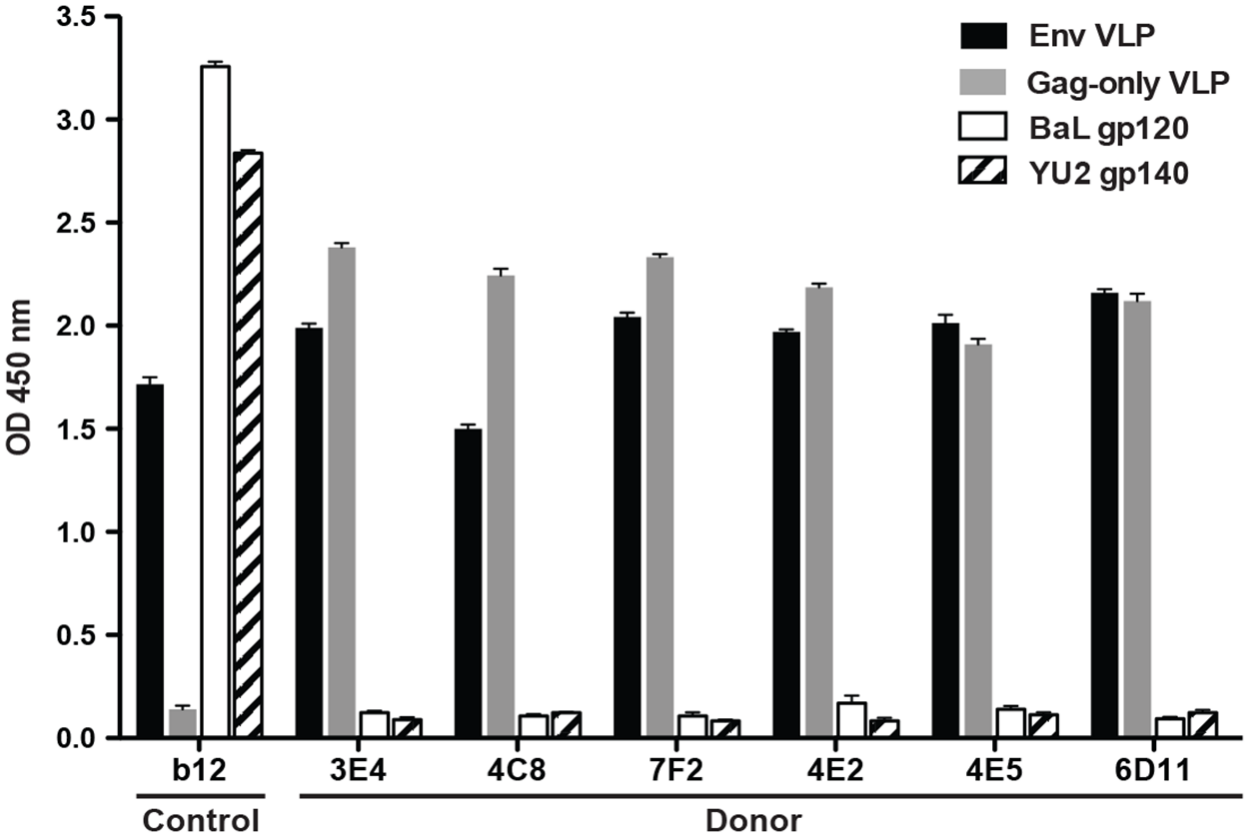
Binding of V_H_4-59 encoded antibodies to HIV antigens. Binding of mAb b12 or V_H_4-59 gene-encoded mAbs at a concentration of 1 μg/mL to Env VLP (black), Gag-only VLP (grey), BaL gp120 (white), or YU2 gp140 (pattern) tested by ELISA.

To characterize the binding phenotype of these V_H_4-59 gene-encoded mAbs, we synthesized genes encoding these mAbs as cDNAs optimized for expression in mammalian cells and expressed recombinant IgGs in 293F cells. The purified IgGs were examined for binding to VLPs displaying envelope (Env-VLP), VLPs lacking Env (Gag-only VLPs), or purified gp120 from the BaL strain (BaL gp120). The most remarkable finding noted was the binding of mAb 3E4 to both Env-VLP and Gag-only VLP, with no binding observed to BaL gp120 protein. This finding showed that mAb 3E4 binding to the VLPs was not Env-specific. As expected, the previously described Env protein CD4-binding site-specific mAb b12 bound to Env-VLP and BaL gp120, but not to Gag-only VLPs. To determine whether this Gag-only VLP binding phenotype was prevalent among five other V_H_4-59 encoded mAbs, we tested binding to Env-VLP, Gag-only VLP, BaL gp120, or YU2 gp140. All the V_H_4-59 gene-encoded mAbs tested showed binding to the VLPs independent of Env, but binding was not observed to purified Env proteins in monomeric or trimeric forms (Fig 2). The binding to Gag-only VLPs but not to purified Env suggested an Env-independent mode of binding to some component of Gag-only VLPs, such as lipids in cell membrane, host proteins incorporated into budded particles, or an HIV protein component of Gag.

### Reactivity of recombinant forms of Gag-only VLP reactive antibodies

**Fig 3:**
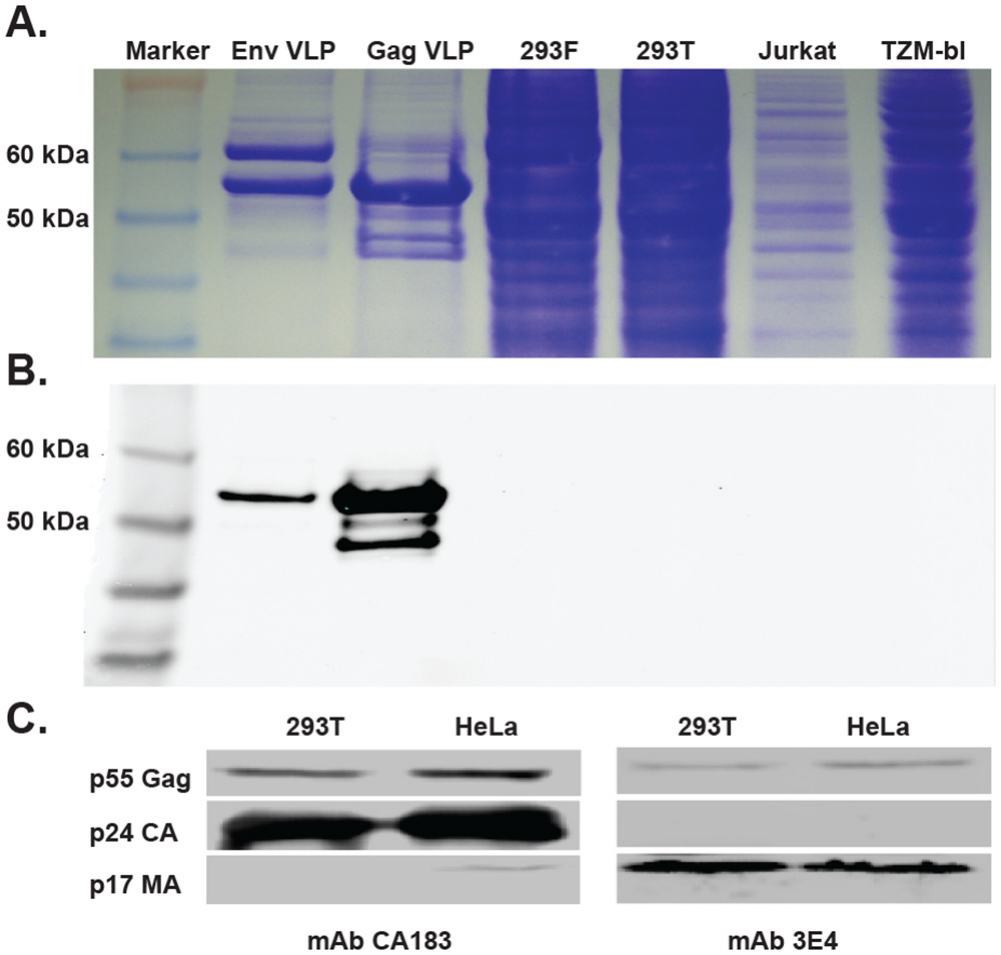
Identifying HIV proteins recognized by mAb 3E4. **(A)** SDS-PAGE separation on a 4–12% gradient gel of Env VLP, Gag-only VLP, 293F, 293T, Jurkat or TZM-bl cell lysates, followed by Coomassie staining. (**B**) Western blot analysis of the cell lysates using mAb 3E4 as a probe. The molecular weight markers are indicated. **(C)** Western blot analysis of NL4-3 pelleted particles using mAbs CA183 (left panel) or 3E4 (right panel) as probes. Bands representing the molecular weights of p55 Gag, p24 CA and p17 MA are indicated.

The occurrence of a single heavy chain variable gene segment encoding antibodies with the Gag-only VLP binding specificity suggested that the binding might not just be nonspecific interaction of diverse B cells to varying components of the VLPs, but rather a specific molecular interaction mediated by antibodies encoded by this gene segment. We sought to determine whether Gag-only VLP binding was due to specific interactions with host proteins from the cell membrane or to HIV internal proteins. We tested binding of mAb 3E4 as a representative mAb of this class to lysates made from several human cell lines or from Gag-only HIV VLPs. Lysates were electrophoresed on two 4–12% NuPAGE Bis-Tris gels, one of which was stained by Coomassie blue staining to verify the presence of proteins in the cell lysates (Fig 3A). In order to determine the size of any proteins in the cell lysates interacting specifically with mAb 3E4, we performed western blot analysis on the second gel after electrophoresis, using mAb 3E4 as a probe. We observed the presence of a band with a 55 kDa apparent molecular weight in the lysates from the VLPs, which was absent in the lysates from the human cell lines (Fig 3B). This finding indicated that mAb 3E4 was binding to a viral component of the VLPs, recognizing a band at an apparent molecular weight of 55 kDa that could be Gag because of the expected migration of p55 Gag. To test our hypothesis, we performed western blot analysis using cell lysates from NL4-3 virus produced in 293T or HeLa cell lines, using mAb 3E4 as a probe. As a positive control, we included capsid-specific mAb CA183 that binds to a region on the top of Gag (Fig 3C, left panel). The results showed that mAb 3E4 binds to p55 Gag and p17 Matrix proteins (Fig 3C, right panel). These findings revealed that the V_H_4-59 gene-encoded mAbs bind to the p17 Matrix protein.

### Recognition of virion particles with varying maturation state

Since these Gag-specific mAbs bound to p17 and to intact HIV-VLPs, we next asked whether they differentially recognized mature versus immature virion particles. We tested binding of one of the mAbs, 3E4, to HIV virion particles using an established whole virion imaging method [27], and did not observe differential binding to mature versus immature virion particles (data not shown). These results suggest that recognition of Gag by mAb 3E4 is independent of late cleavage events that occur during the HIV-1 life cycle.

### Mapping the epitope of mAb 3E4

**Fig 4:**
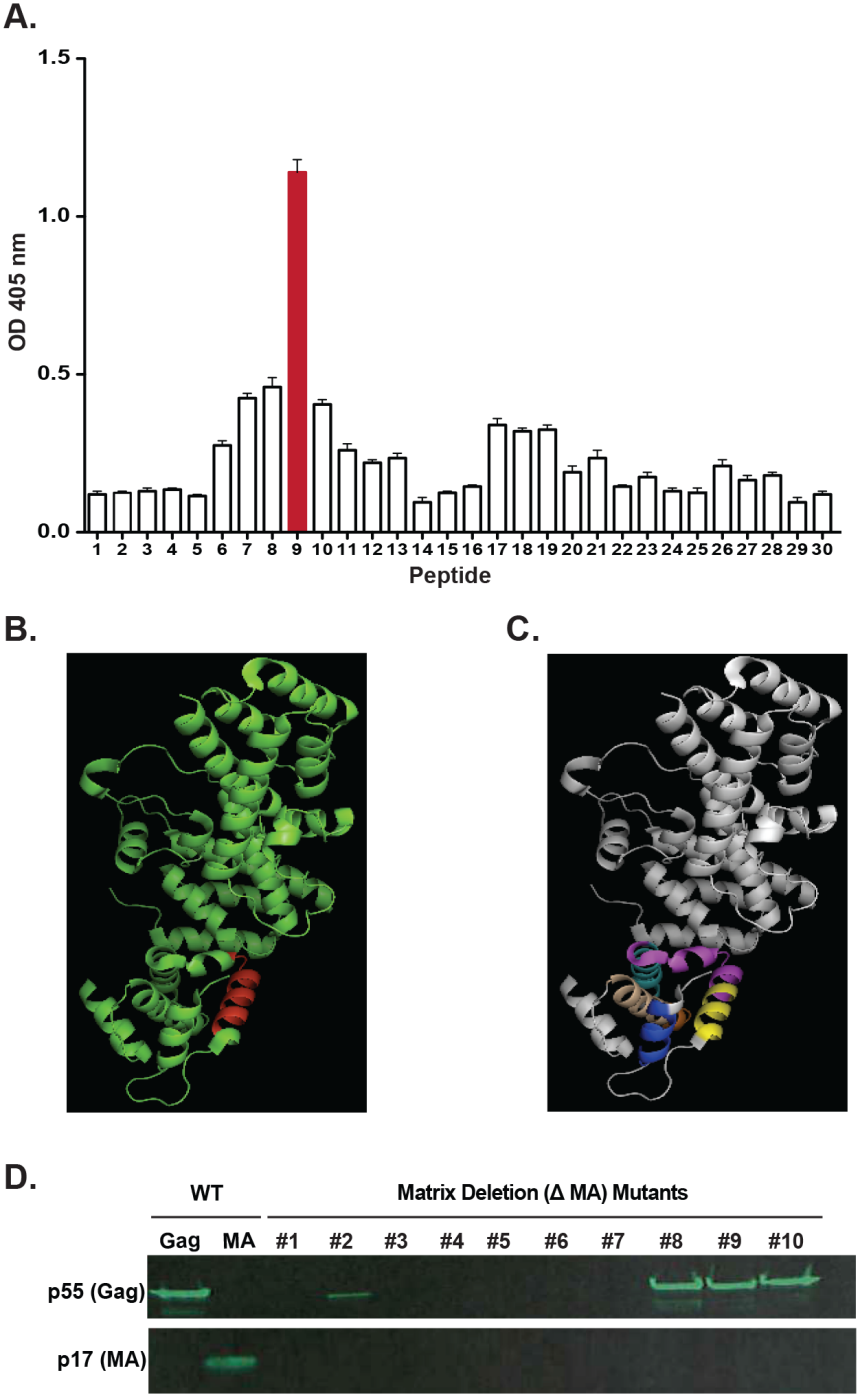
Mapping the mAb 3E4 epitope on the surface of HIV Gag protein. **(A)** Binding of mAb 3E4 to overlapping linear peptides of the HIV-1 Gag protein. **(B)** Structural depiction of the mAb 3E4 epitope (shown in red) on the HIV-1 Gag protein. **(C)** Structural depiction of deletion mutants #1, #3, #4, #5, #6, and #7 (shown in blue, yellow, magenta, cyan, orange or wheat) on the surface of the HIV-1 Gag protein. **(D)** Western blot analysis of Gag, wild-type Matrix protein, or each of 10 different Matrix protein deletion mutants, using mAb 3E4 as a probe.

**Fig 5:**
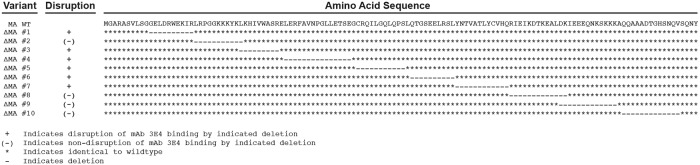
Generation of p17 Matrix deletion mutants. The full amino acid sequence alignment of the wild-type (WT) Matrix protein and the 10 Matrix deletion mutants. Conserved residues in the Matrix deletion mutants are indicated by the * symbol, and deleted resides indicated by the—symbol.

Next, we used a collection of overlapping peptides based on the HIV-1 group M consensus sequence of Gag to map the epitope of mAb 3E4. By testing binding to overlapping linear peptides, we determined that mAb 3E4 bound to a peptide in the p17 region of the Gag protein (Fig 4A). This peptide, with an amino acid sequence of HLVWASRELERFALN, is located on the surface of the Gag protein in the native structure (Fig 4B), exposing it for antigen-antibody interactions. Murine antibodies to this epitope have been described and have been shown to bind the surface of infected cells [28]. Given the alpha helical nature of the region where the peptide was located, we were interested in determining whether mutations in this region would affect the binding of mAb 3E4 to the p17 protein. We hypothesized that mutations in the globular head of the p17 protein would abrogate binding of mAb 3E4, due to a loss of stability associated with breaking alpha helices and the resulting conformational shifts that would occur. We generated ten p17 deletion mutants to test this hypothesis, by making overlapping amino acid deletions in the regions around the mAb 3E4 epitope on the p17 protein (Fig 5). We tested binding of mAb 3E4 to these p17 deletion mutants by western blot analysis, and observed a loss of binding to deletion mutants #1, #3, #4, #5, #6, and #7 shown in blue, yellow, magenta, cyan, orange or wheat colors, respectively (Fig 4C and 4D). These results showed that mutations in the globular head of the p17 protein abrogate the binding of mAb 3E4 to p17.

### Immunologic assays

**Fig 6:**
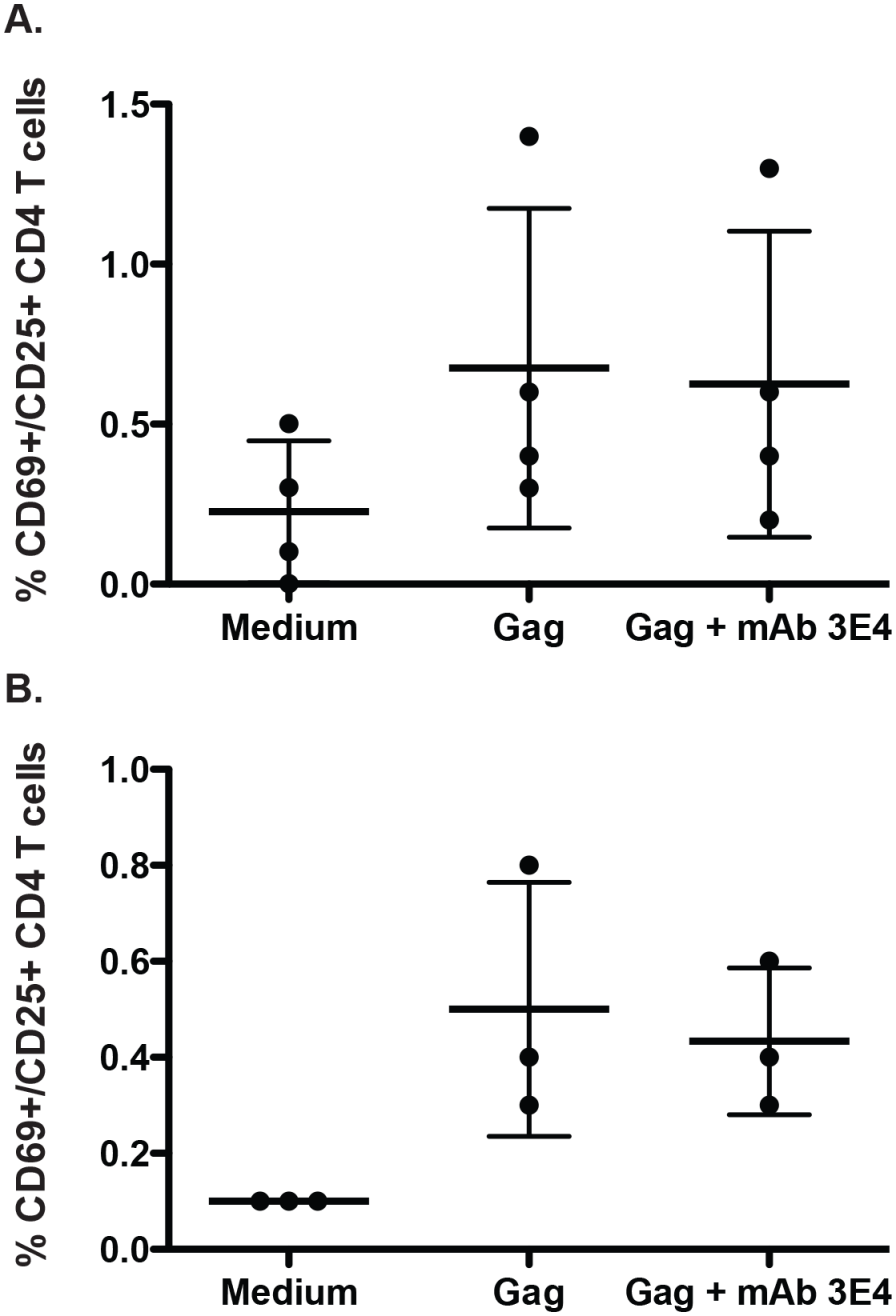
Activation of T cells by Gag in the presence or absence of p17-specific mAb 3E4. **(A)** Activation of Gag-specific T cells from four subjects in the presence or absence of mAb 3E4. **(B)** Activation of Gag-specific T cells from one subject, tested in triplicate.

We next sought to determine if Gag served simply as an antigenic target for these antibodies or, alternatively, if these antibodies mediated some measurable *in vitro* functional activity that might contribute to the functional immune response in this subject. MAb 3E4 did not exhibit any detectable neutralizing activity against the clade B HIV-1 isolate SF162, and we found no evidence of activity in ADCVI assays (data not shown). We also tested whether incubation of mAb 3E4 with Gag protein would enhance (or abrogate) stimulation of Gag-specific T cells in PBMCs from treatment-naïve HIV-infected donors with known Gag responses. There was no detectable enhancement or inhibition of Gag-specific T cell activation in the presence of mAb 3E4 in any of the individuals as measured by changes in CD69 and CD25 expression (Fig 6). Additionally, cytokine assays measuring IFN-γ, IL-2, and TNF-α and additional activation markers including CD86 and CD40L were performed on PBMC from the subject with the most robust Gag responses. In these assays, incubation with Gag protein and serial dilutions of mAb 3E4 did not yield any differences in Gag-specific responses (data not shown).

## Conclusions

Diversity in the B cell response to HIV-1 in infected individuals is driven principally by the immunogenicity of a subset of HIV-1 proteins. The strongest research focus in recent years has been on identification and characterization of broadly neutralizing antibodies that target the HIV-1 Env glycoprotein. The moderate success of the RV144 vaccine trial and the association of ADCC-mediating antibodies as a correlate of protection have generated significant research interest in these kinds of antibodies [29]. There is limited information on the dominant epitopes or antibody genes that drive human B cell response to targets other than Env, however. Here, we identified and characterized a panel of mAbs that recognized the HIV-1 Gag and p17 proteins, and mapped the fine specificity of the epitope for one of them, 3E4, to the globular head of the p17 protein. It seems likely that many or all members of this class of antibody works in a similar fashion to 3E4, although there are several caveats about generalization of the results of the detailed mapping studies here. First, we used a representative antibody for detailed studies, and second we used identical light chains in the recombinant forms of these V_H_4-59 encoded antibodies. The common use of a single V_H_ gene segment in diverse clones with similar phenotype typically suggests a dominant effect of the heavy chain CDR2 loop, but our use of a common light chain may underestimate the modulating effects of light chain diversity in recognition of this epitope.

It was interesting that p17-specific antibodies were common in the response, and the V_H_4-59 variable gene segment encoded all of the p17-specific mAbs isolated in this study. Increasingly, association of antibody genes with antigen- or epitope-specific responses has been reported. In HIV, this V_H_4-59 variable gene segment has been associated with AIDS-associated B-cell lymphoma [30]. We have previously described a strong association of use of the V_H_1-46 gene segment with human antibodies against rotavirus VP6 protein [31]. Use of the V_H_1-69 germline gene is associated with cross-reactive hemagglutinin (HA) stem-binding mAbs against influenza, the influenza HA receptor binding domain, HIV-1 CD4 induced epitopes, hepatitis C virus, and others [32–34]. Antibodies against the V3 domain of HIV-1 Env preferentially use the V_H_5-51 germline gene [35]. The identification here of six Gag-specific mAbs sharing a common V_H_4-59 germline gene in a single HIV-infected individual, suggests that this gene segment may constitute an important component of Gag-specific antibody responses in infected individuals. Typically, such V_H_ gene segment associations indicate a favorable structure of the heavy chain complementarity-determining region 2 (HCDR2), since the V_H_ gene segment encodes the HCDR1 and HCDR2 loops, while the HCDR3 region is encoded by the V_H_-D-J_H_ junction. Understanding such antibody gene associations with epitope specificity is increasingly valuable as investigators seek to deconvolute the significance of over-represented antibody sequences in next generation sequencing studies of the antibody repertoires in humans following vaccination or infection [36].

The presence of p17 serum antibodies sometimes correlates with the presence of HIV-1 neutralization [37]. We tested the functional activity of one of these V_H_4-59 encoded antibodies, 3E4, extensively and found no evidence that this mAb mediated any significant immunologic function *in vitro*. Thus, it is likely that the immunodominant epitope in p17 that we identified here distracts the B cell response from more functional targets. Releasing large amounts of highly immunogenic HIV-1 proteins like Gag, with the induction of high levels of functionally irrelevant antibodies could contribute to the delayed development of neutralizing antibodies in infected individuals.
